# Mycotoxins Exposure of French Grain Elevator Workers: Biomonitoring and Airborne Measurements

**DOI:** 10.3390/toxins13060382

**Published:** 2021-05-27

**Authors:** Sophie Ndaw, Aurélie Remy, Danièle Jargot, Guillaume Antoine, Flavien Denis, Alain Robert

**Affiliations:** 1 Toxicology and Biomonitoring Department, INRS—French National Research and Safety Institute for the Prevention of Occupational Accidents and Diseases, 54500 Vandoeuvre-Lés-Nancy, France; aurelie.remy@inrs.fr (A.R.); guillaume.antoine@inrs.fr (G.A.); flavien.denis@inrs.fr (F.D.); alain.robert@inrs.fr (A.R.); 2 Pollutant Metrology Department, INRS—French National Research and Safety Institute for the Prevention of Occupational Accidents and Diseases, 54500 Vandoeuvre-Lés-Nancy, France; daniele.jargot@inrs.fr

**Keywords:** mycotoxins, exposure assessment, biomonitoring, air, dust, HR-MS/MS, occupational exposure, grain workers

## Abstract

It is now recognized that additional exposure to mycotoxins may occur through inhalation of contaminated dust at a workplace. The aim of this study was to characterize the multi-mycotoxin exposure of French grain elevator workers using biomonitoring and airborne measurements. Eighteen workers participated in the study. Personal airborne dust samples were analyzed for their mycotoxin concentrations. Workers provided multiple urine samples including pre-shift, post-shift and first morning urine samples or 24 h urine samples. Mycotoxin urinary biomarkers (aflatoxin B1, aflatoxin M1, ochratoxin A, ochratoxin α, deoxynivalenol, zearalenone, α-zearalenol, β-zearalenol, fumonisin B1, HT-2 toxin and T-2 toxin) were measured using a liquid chromatography–high-resolution mass spectrometry method. Grain elevator workers were highly exposed to organic airborne dust (median 4.92 mg.m^−3^). DON, ZEN and FB1 were frequent contaminants in 54, 76 and 72% of air samples, respectively. The mycotoxin biomarkers quantified were DON (98%), ZEN (99%), α-ZEL (52%), β-ZEL (33%), OTA (76%), T-2 (4%) and HT-2 (4%). DON elimination profiles showed highest concentrations in samples collected after the end of the work shift and the urinary DON concentrations were significantly higher in post-shift than in pre-shift-samples (9.9 and 22.1 µg/L, respectively). ZEN and its metabolites concentrations did not vary according to the sampling time. However, the levels of α-/β-ZEL were consistent with an additional occupational exposure. These data provide valuable information on grain worker exposure to mycotoxins. They also highlight the usefulness of multi-mycotoxin methods in assessing external and internal exposures, which shed light on the extent and pathways of exposure occurring in occupational settings.

## 1. Introduction

Grain elevator workers may be exposed to large amount of grain dust during tasks including reception of grain, inspection of elevator or dryer, grain loading/unloading, grain rotation, shipment and cleaning [1]. Grain dust is a mixture of plant fragments, microorganisms, inorganic compounds and fungal particles. Exposure to grain dust is associated with respiratory symptoms [2]. An additional potential risk for these workers is posed by the mycotoxins occurring in grain.

Mycotoxins are secondary metabolites of fungi that are known to exert a wide range of toxicities in humans and animals. Depending on the type of mycotoxins, nephrotoxicity, cancer, liver toxicity, impaired immunological functions and growth retardation have been reported among their adverse health effects [3]. Mycotoxins contaminate many of the most frequently consumed foods and feeds worldwide, including cereals, nuts, dried fruits and spices. Ingestion of contaminated foodstuffs results in a dietary exposure of the general population, while high exposure to organic dust can be a source of exposure for workers via inhalation or through dermal contact. Studies have shown that the amount of mycotoxins found in dust can be more than 10 times greater than that found in raw materials. Indeed, mycotoxins are largely present on the surface of raw materials and tend to be adsorbed into the dust during handling [4,5].

Exposure of workers to grain dust should therefore be investigated: firstly, to determine the extent to which workplace exposure compares with exposure resulting from ingestion of mycotoxin-contaminated food, and secondly, to evaluate the magnitude of the potential health risk due to occupational exposure to mycotoxins.

Viegas et al. [6] have performed an extensive review of the studies on occupational exposure to mycotoxins. A limited number of studies have reported the prevalence of mycotoxins in airborne dust and settled dust samples in grain industries, including commercial seaports, grain elevators and feed mills [4,5,7,8,9,10,11]. Biomonitoring studies in grain industries have focused on occupational exposure to aflatoxins [11,12,13,14] and OTA [15], mainly in blood samples. It is, however, known that the co-occurrence of mycotoxins in food and feed is common [16,17,18], and humans are often exposed to more than one mycotoxin at the same time. Only Follmann et al. [19] have reported the use of a multi-biomarker approach among grain workers to assess exposure to mycotoxins in urine samples.

Urine is a convenient matrix for biomonitoring studies and has been shown to be suitable for the determination of biomarkers of most of the mycotoxins of toxicological relevance [20,21,22,23,24]. In order to interpret correctly the exposure results, the use of urine samples in occupational studies requires careful consideration of the sampling strategy and the nature of biomarkers to be analyzed [20]. Information on toxicokinetics of the mycotoxins should be taken into account for this purpose when performing biomonitoring studies.

The aim of this study was to characterize multi-mycotoxin exposure of grain elevator workers in France. Airborne mycotoxin levels were studied concurrently with mycotoxin biomarker urinary concentrations in workers. The methodological design was carefully considered. The biomonitoring sampling strategy consisted of the collection of 24 h urine samples or the collection of multiple spot urine sample over several days. Quantitative data were collected using biomonitoring and airborne measurements, validated in a previous pilot study [25]. The mycotoxins selected—aflatoxin B1 (AFB1), ochratoxin A (OTA), zearalenone (ZEN), fumonisin B1 (FB1), T-2 toxin (T-2) and HT-2 toxin (HT-2)—were considered to be among the mycotoxins of greatest importance and were quantified in personal airborne dust samples. Relevant urinary biomarkers, such as AFB1, aflatoxin M1 (AFM1), OTA, ochratoxin α (OTα), DON, ZEN, α- and β-zearalenol (α-and β-ZEL), FB1, HT-2 and T-2, were measured with a multi-class mycotoxin method based on liquid chromatography–high-resolution mass spectrometry (LC-HRMS).

## 2. Results

### 2.1. Study Population

Eighteen male workers were recruited for this study. They were investigated during the wheat and maize harvests in France, which are the busiest periods of the year. From grain elevator A, there were four workers during the wheat harvesting period in July (grain elevator A1) and eight workers during the maize harvesting period in October (grain elevator A2). Six additional workers were included from grain elevator B. The characteristics of the study population are summarized in Table 1. The median age was 44 years in the grain elevator A group and 36.5 years in the grain elevator B group. The number of years as a grain worker was highly variable and ranged from less than 1 month to 35 years. Sixty-six percent of workers were active smokers. The analysis of the questionnaires for cereal-based food consumption did not show noticeable differences between workers.

During a full shift, grain workers were involved in one or more tasks including reception of grain, inspection of elevator or dryer, grain loading/unloading, grain rotation, shipment and cleaning. FFP2 respiratory protection masks were worn occasionally throughout the shift. Wheat was the main grain processed in elevator A1 while maize was mostly handled in elevators A2 and B. In addition to wheat or maize, the participants reported handling rye, barley, triticale and oleaginous seeds as well, but to a lesser extent.

### 2.2. Exposure Level to Grain Dusts and Mycotoxins in Air Samples

Personal airborne dust samples were collected for three consecutive days. The duration of collection varied between 5 and 9.5 h, depending on the work shift. The mean sampling duration was 7.5 h. The sampled air volume collected was between 3.05 and 5.91 m^3^. The samples were analyzed for the presence of seven mycotoxins: DON, ZEN, FB1, AFB1, OTA, T-2 and HT-2. Table 2 presents the dust concentrations and quantified mycotoxin levels in air samples for the three elevators.

Dust concentrations in personal air samples ranged from non-detected to 43.4 mg.m^−3^ in all settings. The median concentration was 4.2 mg.m^−3^ in elevator A1 and A2, and it was higher, 7.9 mg.m^−3^, in elevator B.

Airborne dust samples showed quantifiable levels of DON, ZEN, FB1, T-2 and HT-2. AFB1 and OTA were not quantified in any sample, probably because they were mainly produced post-harvest. Mycotoxin occurrence was variable, depending on the harvest period, i.e., on the variety of grain handled. In grain elevator A1, during wheat harvest, only three samples were positive for mycotoxins among the twelve samples collected. In one sample, three mycotoxins—DON, ZEN, FB1—were quantified. Detectable amounts of DON, ZEN and FB1were measured in 17, 25 and 8% of samples, respectively.

Mycotoxin occurrence was higher in airborne dust collected during the maize harvest in elevators A2 and B. DON was quantified in 64% of samples in elevator A2 and in 69% of samples in elevator B, while ZEN was quantified in 91% and 94% of samples, respectively. Air dust also showed contamination by FB1 in 86% and 100% of samples collected from elevators A2 and B, respectively. In addition, samples were found positive for HT-2 and for T-2, to a lesser extent. The occurrence of HT-2 was higher in elevator B than in elevator A2. More than 94% of air samples collected during the maize harvest season were contaminated by 3–5 mycotoxins.

Mycotoxin concentrations were at ng.m^−3^ level in air samples (Table 2). Concentrations were registered between LOQ–80.1 ng.m^−3^ for DON, LOQ–778 ng.m^−3^ for ZEN, LOQ–248 ng.m^−3^ for FB1, LOQ–417 ng.m^−3^ for T-2 and LOQ–2232 ng.m^−3^ for HT-2. For the same harvesting period, mycotoxins were quantified in lower amounts in elevator A2 than in elevator B. Median concentrations (Table 2) varied from 8.3 to 11.0 ng.m^−3^ for DON, 11.0 to 79.5 ng.m^−3^ for ZEN, 13.8 to 28.6 ng.m^−3^ for FB1 and 83.9 to 406 ng.m^−3^ for HT-2. The mycotoxin HT-2 was detected in higher levels than any other one.

Mycotoxin respiratory intake was estimated at median concentrations in air samples in all settings. An average volume of breathed air of 30 L per minute for moderate physical activity was considered [26]. Estimated inhaled dose during 8 h work was 115 ng, 173 ng and 189 ng for DON, ZEN and FB1, respectively.

### 2.3. Biomonitoring Data

#### 2.3.1. Mycotoxin Biomarkers in Urine Samples

A total of 195 urine samples were collected from eighteen workers. The biomonitoring data are reported in Table 3. Mycotoxin biomarkers were analyzed and quantified as their “total” forms (aglycone plus conjugates), after an enzymatic hydrolysis step. Samples with mycotoxin concentrations above the respective limits of quantification LOQ were considered as positive. The mycotoxin biomarkers quantified in urine samples from all settings, expressed as a percentage of positive samples, were DON (98%), ZEN (99%), α-ZEL (52%), β-ZEL (33%), OTA (76%), T-2 (4%) and HT-2 (4%). Mycotoxin biomarkers were more frequently quantified from workers in elevators A1/A2 than in elevator B. This finding was not consistent with mycotoxin occurrence in airborne dust, which was higher in samples collected from elevators A2 and B. The mycotoxins AFB1, AFM1, FB1 and OTα were not quantified in urine samples.

DON was a predominant mycotoxin, quantified at higher level in nearly all samples. Median concentration for all workers was 14.50 µg/L (12.40 µg/g crea) and varied from 13.30 to 16.80 µg/L within the three elevators. DON concentrations ranged from LOQ to 154 µg/L.

ZEN was also a predominant mycotoxin that was quantified in almost 100% of samples. Median concentration for all workers was 0.13 µg/L (0.11 µg/g crea). ZEN metabolites α-ZEL and β-ZEL were found in few samples and mainly in urine samples of participants working in elevators A1 and A2. α-ZEL median concentrations were 2.16 µg/L (1.11 µg/g crea) in elevator A1 and 1.73 µg/L (1.26 µg/g crea) in elevator A2. The highest concentrations of α-ZEL were quantified in the urine samples from a worker in elevator A1. The maximum concentration of 48.9 µg/L was measured on day 2. However, his personal air sample did not feature high ZEN concentration (6.97 ng.m^−3^). The highest β-ZEL concentrations (up to 3.72 µg/L) were also quantified in the urine samples from the same worker. β-ZEL median concentration, 0.90 µg/L (0.44 µg/g crea), could only be calculated for samples collected in elevator A1, as the proportion of measurements below the LOQ was >50% in elevator A2 and B.

OTA was quantified at lower concentration, compared with DON and ZEN. The median concentrations calculated in the different settings ranged from 0.013 to 0.025 µg/L (0.009–0.017 µg/g crea). The percentage of positive samples was higher (94%) in elevator A2 than in elevator A1 (80%) and B (58%).

T-2 and HT-2 were far less quantified in urine samples. Positive samples to T-2 were found in elevator A1/A2 (5–12%) and in elevator A1 for HT-2 (10%). Concentrations were between LOQ–2.65 µg/L and LOQ–5.63 µg/L for T-2 and HT-2, respectively. While the highest airborne concentrations of T-2 and HT-2 were measured in elevator B, T-2 and HT-2 were not quantified in urine samples from the workers of this elevator. One of the reasons may be a more systematic use of respiratory protective masks.

When compared with external exposure, biomarkers of the mycotoxins that were quantified in air samples (DON, ZEN, FB1, T-2, HT-2) were quantified in urine samples as well, except for FB1. OTA was not detected in air samples; thus, it can be assumed that workers were not occupationally exposed to OTA.

Subsequent statistical analysis in this study was then restricted to DON and ZEN biomarkers, as the proportion of measurements below the LOQ was too high for T-2 and HT-2.

#### 2.3.2. Urinary DON Excretion Profile in 24 h Urine Samples

**Figure 1:**
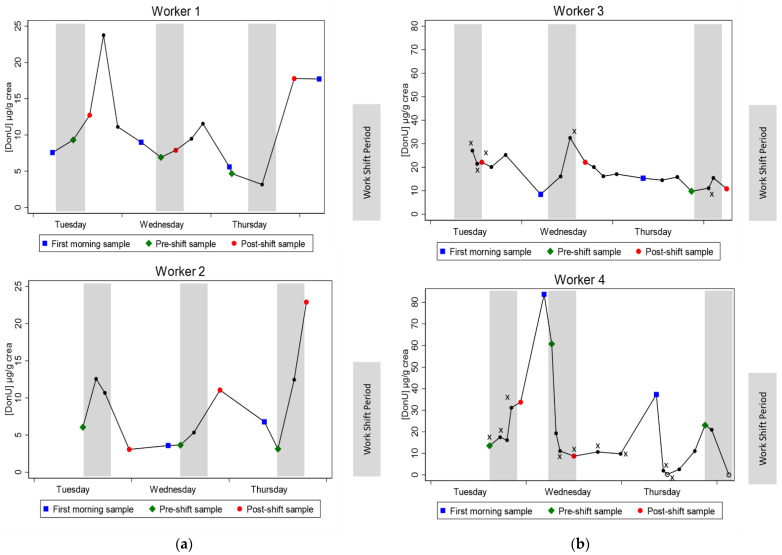
Individual kinetic profiles of urinary DON excretion over the sampling week. Plot of urinary DON concentration (µg/g) versus time for (**a**) workers from elevator A1 (worker 1 and worker 2) and (**b**) workers from elevator B (worker 3 and worker 4). Plain bars represent the work shift periods. x represents samples in which creatinine value is out of range ((creat) < 0.5 g/L or >3 g/L). Number of years of activity: 15 (worker 1), 9 (worker 2), 17 (worker 3), 4 (worker 4).

The elimination kinetic profile of DON was assessed in 24 h urines samples collected from volunteers of the grain elevator A1 and grain elevator B. The objective was to visualize any increase in urinary DON concentration after a work shift and to figure out a preferred sampling period for assessing the occupational exposure. Ten participants provided 24 h urine samples for three consecutive days, from Tuesday to Friday. The number of samples collected was between 3–7 samples/24 h/participant. Participants did not report any missed samples. However, workers with a low number of samples (<4 samples/24 h) were not further included to assess the elimination kinetic profile of DON. Figure 1 shows the urinary DON concentrations (µg/g crea) for four workers over three days, each graph representing data for an individual worker. The plain bars across the graphs represent the work shift periods for each working day. The urine samples that were collected as close to the start and end of the work shift as possible were labeled pre- and post-shift samples, respectively.

DON elimination profiles exhibited high variations in urinary concentration over 24 h. The highest concentrations were mostly observed in samples collected after the end of the work shift (Figure 1a,b). The high concentrations could be attributed to an exposure during the shift. However, urinary excretion peaks were also noticed for worker 4 in elevator B who had more than 24 h off between two work shifts on Wednesday and Thursday (Figure 1b). Such an increase could also be attributed to non-occupational sources. It is worth noting that half of the urine samples collected from worker 4 had out of range ((creat) < 0.5 g/L or >3 g/L) creatinine concentrations. Thus, DON concentrations in these samples may be less accurate.

The amount of DON excreted per day (expressed in μg/24 h) was calculated for the ten workers who provided 24 h urine samples. The median was 20.10 μg/24 h, the amounts ranging from 8.90 to 53.60 μg/24 h.

#### 2.3.3. Comparison of DON, ZEN and OTA Biomarker Levels at Different Sampling Times

Mycotoxin (DON, ZEN, OTA) biomarker concentrations for pre-shift, post-shift and first morning urine samples are shown in Table 4 and Table 5.

Variations in DON median concentrations were observed, depending on the sampling time. The difference was not significant between pre-shift and first morning urine samples for DON exposure in the three groups. However, concentrations of DON appeared to be higher in post-shift samples than in pre-shift samples. The difference was significant for grain elevator A1 (pre-shift median concentration of 6.71 µg/L and post-shift median concentration of 26.70 µg/L; *p* = 0.001) and grain elevator A2 (pre-shift median concentration of 13.10 µg/L and post-shift median concentration of 24.80 µg/L; *p* = 0.021). The difference obtained for grain elevator B was not significant (*p* = 0.497). Statistical analysis performed with values adjusted for creatinine (Table 5) for all settings led to the same conclusions: urinary DON concentrations were higher in post-shift samples than in pre-shift-samples.

For ZEN, no difference was observed in urinary concentration according to the sampling time, except for post-shift sample levels that were higher than first morning samples levels for the values adjusted for creatinine (Table 5) for all settings (*p* = 0.004). The median concentration varied from 0.13 µg/g to 0.09 µg/g. However, any conclusion related to this difference would be hazardous. Indeed, ZEN metabolites, α-ZEL and β-ZEL, were largely more excreted than ZEN (at least 10 times) in this study, and no difference was found in α-ZEL concentrations according to the sampling time (Table 4). Statistical analysis was not performed for βZEL due to the high number of samples with concentration below the LOQ.

The statistical analysis of OTA data did not feature any difference between pre-shift, post-shift and first morning sample concentrations.

### 2.4. Linking Biomonitoring Data to External Exposure

**Figure 2:**
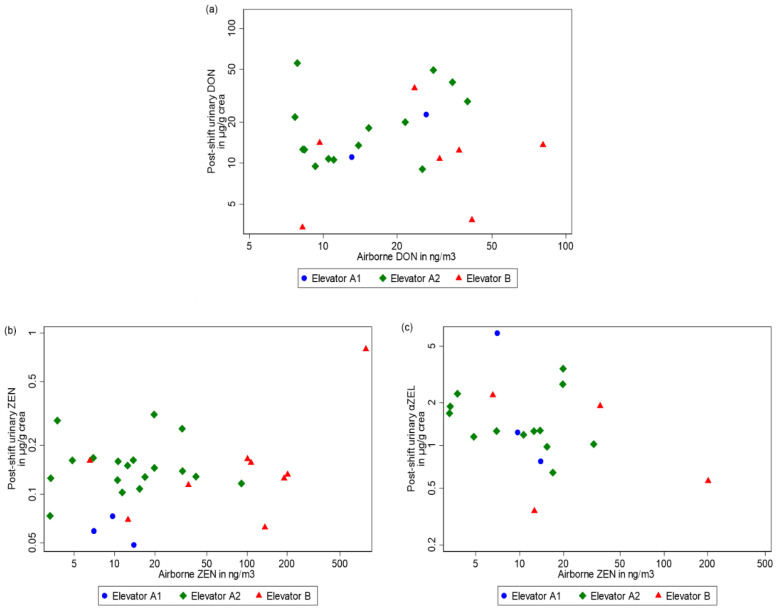
Scatter plot showing post-shift urinary (**a**) DON, (**b**) ZEN and (**c**) α-ZEL concentrations (µg/g) versus airborne mycotoxin concentrations.

The relation between DON and ZEN levels in air samples and the mycotoxin biomarker levels in urine samples was tested, using the Pearson correlation coefficient on the log-transformed urinary and airborne mycotoxin concentrations. Figure 2 represents the plot of post-shift urinary biomarker concentrations as a function of airborne mycotoxin concentrations, when mycotoxins were quantified in both samples.

The correlation coefficients were low for DON, ZEN and α-ZEL (*r* = −0.17, *r* = 0.014, *r* = −0.58, respectively). No correlation was found between atmospheric and urinary DON, either between atmospheric and urinary ZEN or α-ZEL. Urinary mycotoxin concentrations were not correlated with the external atmospheric exposure measured.

## 3. Discussion

The nature of mycotoxins and the magnitude of exposure can vary depending on the workplace, the products handled or the tasks performed by workers. All the participants included in this study were grain elevator workers from the same administrative region in France. They were investigated during the wheat and maize harvesting period. This protocol ensured that the exposure groups were similar for the study.

The grain elevator workers were highly exposed to organic airborne dust. The median concentration of all samples (*n* = 50) was 4.92 mg.m^−3^ during their shift. The protocol implemented allows a good estimate of inhalable dust intake given that the personal air samples were collected during nearly the whole shift. Data on airborne exposure in grain industries is very limited. Mayer et al. [7] reported a mean airborne dust concentration of 3 mg.m^−3^ in grain elevators, but information on the sampling duration was lacking. Halstensen et al. [27] collected personal air samples (*n* = 96) from 84 farms. Sampling durations were between 6 and 60 min. The concentration of total inhalable dust estimated varied from 0.2 to 108 mg.m^−3^, and the median was 5 mg.m^−3^. In a survey performed among feed mill workers, Ferri et al. [11] reported mean concentration up to 9.4 mg.m^−3^ from pooled personal air samples.

The investigation on the mycotoxin presence in airborne dust showed that DON, ZEN and FB1 are frequent contaminants in grain elevators in France. They were quantified in 54%, 76% and 72% of all samples, respectively. Even though differences in incidence were observed between elevators, airborne dust samples were usually contaminated by more than two mycotoxins. Similar observations have been reported in Switzerland [8]. Quantifiable levels of DON, ZEN and nivalenol have been found in full shift personal airborne samples collected during wheat harvest and grain handling. The highest mean concentrations reported were 64.7 ng.m^−3^ for DON and 3.3 ng.m^−3^ for ZEN, at the same magnitude as the concentrations found in the present study. Contamination of settled dust by multiple mycotoxins including DON, OTA and ZEN was also reported in grain elevators in Norway [5], in Germany [7] and in storage facilities in Belgium [9]. However, OTA was not detected in air samples collected during this study, suggesting that grain elevator workers were not occupationally exposed to this mycotoxin during the periods of harvest.

All these findings confirm that grain dust could be a source of mycotoxin exposure for workers. Respiratory intake could be estimated from the mycotoxin median concentrations in airborne samples. Estimated inhaled doses for 8 h work in this study were 115 ng, 173 ng and 189 ng for DON, ZEN and FB1, respectively. Those estimated doses appear to be below their respective provisional maximum tolerable daily intake in food (1, 0.25 and 2 µg/kg b.w.) even if they are not directly comparable [28].

One still open question is whether the airborne dust collected is fully representative of the respiratory intake. Inhalable aerosol sampler collection efficiency is generally low for particles with aerodynamic diameters < 3 µm [29,30]. Several authors [7,31] have suggested that the respirable grain dust contains more mycotoxins than the inhalable dust fraction. Olenchock et al. [32] reported that the largest fraction of particle size distribution in settled dust (originated from airborne dust) is ≤5 µm. These statements suggest that mycotoxin respiratory intake may be underestimated in grain industries when inhalable fraction is collected. Furthermore, the contamination of dust samples by modified mycotoxin forms of DON and ZEN [28] has not been investigated in this study. These forms, if present, could add to the overall burden. In addition, respiratory masks that were worn occasionally, may contribute to a reduction of exposure. All of these points, in addition to dietary exposure, may have led to the low correlation between urinary mycotoxin concentrations and the external atmospheric exposure measured in air samples.

Biomonitoring is a valuable tool for assessing the overall exposure to mycotoxins of workers by all routes: inhalation, dermal and/or ingestion due to hand–mouth contact. This requires knowledge regarding the toxicokinetics of mycotoxins via different exposure routes to define relevant biomarkers and the ideal sampling time. Urine matrix has been frequently used to assess mycotoxin exposure in both the general population and occupational studies [11,19,21,22,23,24,33,34,35]. Vidal et al. [20] performed a comprehensive review on the biomarkers of exposure related to most commonly investigated mycotoxins, confirming the suitability of the urine matrix for the assessment of exposure to DON, ZEN, AFB1, OTA, T-2, HT-2 and FB1. However, the urine sampling time is of great importance for exposure assessment. It allows researchers to recognize what the workplace environment might be adding to the exposure already occurring because of food intake. Knowledge regarding the toxicokinetics of mycotoxins following exposure via inhalation or through dermal contact in the workplace are lacking. In addition, the assessment of multi-mycotoxin exposure does not allow the implementation of a specific sampling strategy for each individual mycotoxin. As an example, DON is known to be rapidly metabolized after ingestion, and a large amount was excreted within the first six hours [20,36]. On the other hand, animal studies have suggested that ZEN and its main metabolites, α-ZEL and β-ZEL, are eliminated relatively slowly from the tissues by enterohepatic circulation [37,38]. Therefore, defining an ideal sampling time for a multi-mycotoxin exposure study appeared difficult. The sampling strategy in previous occupational studies was not always clearly discussed. A single spot urine sample was usually collected during the shift [19,34,35,39], introducing high variability into the data given the multiple sources of exposure. Our sampling strategy was to include more than one single spot urine sample. Multiple urine samples were collected over 3 days, with a standardized schedule including pre-shift, post-shift and first morning urine samples or with a 24 h urine sampling procedure. That strategy made it possible to overcome the uncertainties about toxicokinetics and to strengthen the biomonitoring data.

DON, ZEN and OTA were the predominant mycotoxins. One limitation of this study was that controls were not included for evaluating the contribution of the workplace environment in the total exposure. The main reason was that the possible exposure of controls from the occupational settings investigated could not be excluded due to the presence of dust in the overall working environment. Biomarker levels can however be compared with data for European adults available in other studies.

The profile of urinary DON elimination obtained from workers highlighted the high variations in urinary concentrations over 24 h. The data suggest a rapid urinary excretion and variable exposure sources for DON. Total DON median concentration was 10.3 µg/L from an Italian cohort [40], 3.37 µg/L (mean) from Swedish adults [41] and 10.1 µg/g (geometric mean) from UK adults [42]. In a study on mill workers, Follman et al. [19] reported median levels of 6.5 µg/L, while the median was 6.8 µg/L for farmers in France [39]. The values observed in our study indicated higher DON exposure for grain elevator workers, with a median concentration of 14.50 µg/L (12.40 µg/g crea). In addition, the higher level in post-shift samples suggests an influence of occupational exposure in the body burden of DON.

European biomonitoring data for ZEN is limited. α-ZEL was found in only one sample from a cohort of 239 Belgian adults [43]. ZEN and its metabolites were quantified among 252 adults in Sweden [41]. Less than 40% of samples were positive, at mean levels of 0.03, 0.03 and 0.02 µg/L for ZEN, α-ZEL and β-ZEL, respectively. Solfrizzo et al. [40] reported a median level from the general population (*n* = 52) of 0.056 µg/L for ZEN, and α-/β-ZEL were detected in almost all samples at median levels of 0.074 and 0.088 µg/L. More recently, Ali et al. [21] reported median concentration in German adults (*n* = 60) of 0.07, 0.130 and 0.03 µg/L for ZEN, α-ZEL and β-ZEL, respectively. ZEN biomarker levels were lower among mill workers (*n* = 13) [19]. ZEN was detected in 100% of samples (median of 0.037 µg/L), α-ZEL in 33% of samples (median of 0.005 µg/L) and β-ZEL in 17% (median of 0.005 µg/L). In our study, ZEN median concentration was 0.13 µg/L, higher than the previously reported data in general population and occupational cohort. In addition, α-ZEL median concentration (and β-ZEL level at some point) was almost 10 times higher than ZEN concentration (median of 1.17 µg/L). It confirms that α-ZEL is a predominant biomarker for ZEN and suggests high exposure of grain elevator workers. The low variation of ZEN and α-ZEL concentrations at different sampling time would support a slow elimination rate [38]. To our knowledge, it is the first time that such findings have been reported. Further investigations are clearly needed to confirm and refine these data and to obtain a reliable pattern of ZEN excretion using an analytical method more sensitive than the one used in our study.

Given that OTA was not detected in airborne samples, it was concluded that occupational exposure to OTA was unlikely in this study. Since OTA was quantified in more than 75% of urine samples, its presence was probably related to the consumption of food commodities. Urinary OTA concentrations were very low (median of 0.018 µg/L) and similar to concentrations reported from Belgian adults (median of 0.015 µg/L) [43]. Higher level of OTA was reported in samples from the general population in Italy (median of 0.061 µg/L) and Germany (median of 0.14 µg/L) [40,44]. Among mill workers [19], the median concentration of OTA was 0.091 µg/L, and there appears to be no significant difference between workers and controls. OTA was also detected in samples from workers of a fresh bread dough company in Portugal, but the concentrations were below the LOQ [34]. Ali et al. [44] have reported that OTα may serve as an additional indicator, as its incidence was 78% in their cohort. However, OTα was not detected in our study.

While quantified in airborne samples, mainly during the maize harvest, FB1 was not quantified in urines samples. In fact, fumonisins have been rarely quantified in samples from a European cohort. Incidence and mean level in urine have been low, for example, 0.004 µg/L and 0.055 µg/L [40,41], probably due to the low excretion of FB1 in urine.

Human biomonitoring for T-2 and HT-2 toxins exposure is still in its infancy, and data on exposure are nonexistent. No T-2 or HT-2 was found in urine samples from adults in Belgium or Germany [43,45]. Low incidence (14%, *n* = 22) was reported in Spanish adults [46], with high mean levels between 13.9 and 14.5 µg/g creat. In our study, T-2 and HT-2 were observed both in airborne dust samples and in some urine samples. To our knowledge, this is the first time that the occurrence of T-2 and HT-2 has been reported in the working environment. Overall, multi-mycotoxin occurrence was stated in all urine samples in this study.

The strength of the present investigation is that airborne mycotoxin levels were studied concurrently in the workplaces of workers whose urine samples were analyzed for their mycotoxin biomarker concentrations. This approach has been proven to be highly relevant and was validated in a previous pilot study [25]. Despite the low number of workers investigated and the lack of a control group, it has been shown that additional exposure to mycotoxins is likely to occur in grain elevators.

Monitoring airborne mycotoxins has made it possible to identify which mycotoxins are present and to understand the exposure scenario in the workplace. However, this sole approach is not sufficient for determining the extent of occupational exposure related to food intake. Our study has demonstrated the usefulness of a biomonitoring tool and has revealed exposure to mycotoxin mixture. It confirms the need for multi-mycotoxin methods in assessing external and internal exposure among workers. Monitoring external exposure should also include the assessment of dermal intake, which has been less investigated. Thus, environmental and biomonitoring studies should be developed to shed light on the extent and pathways of exposure occurring in specific occupational settings. This is of particular interest for risk assessment and for a better implementation of risk management measures. In addition, research regarding the toxicokinetics of mycotoxin following inhalation and dermal exposure are needed in order to implement more accurate biomonitoring studies.

## 4. Materials and Methods

### 4.1. Field Study

The field study was conducted in France in two grain elevators, A and B, between July and October 2017. The grain elevators were located in the northeastern region of the country. The collection sites were port grain elevators with a capacity of storing up to 250,000 tons. The cereals were collected from smaller silos for drying and storage before shipment. Grain workers were informed about the investigation and gave their written consent before inclusion in the study. The internal ethical committee approved the study. Eighteen male workers were included. Workers from grain elevator A were investigated during the wheat harvesting period in July (grain elevator A1) and during the maize harvesting period in October (grain elevator A2). Workers from grain elevator B were investigated during the maize harvesting period in September. Maize and wheat were the predominant cereals handled in the grain elevators, depending on the harvesting period. Rye, barley, triticale and oleaginous seeds such as rapeseed or soy were also handled, but to a lesser extent. Both grain elevators A and B were located in the same administrative region.

Full shift personal airborne dust samples were collected from the workers’ breathing zones, outside of the mask when worn, with a CIP10 personal aerosol sampler (Tecora, Fontenay Sous Bois, France) designed and patented by INRS. The CIP10 was equipped with a rotating filter cup containing a pre-weighted polyurethane collecting foam and a particle size selector for the inhalable aerosol fraction (CIP10-I sampling unit). The airflow was 10 L.min^−1^. Before use, each sampler was calibrated on a test rig using pressure drop compensation, and its stability was estimated by checking the cup rotation speed with an ARC 8527 tachometer (Tecora, Fontenay Sous Bois, France). The flow rate was measured and recorded before the beginning of sampling. Air sampling was interrupted during lunch break and replaced once the lunch break was finished. During the break, the flow rate was checked, recorded, then adjusted if necessary. Pre-lunch sampling period was also recorded. At the end of the work shift, the flow rate and the sampling period were once again recorded. The overall sampling duration was the sum of pre-lunch and post-lunch sampling periods. The sample air volume was calculated by multiplying the average flow rate for each period by its duration. The foam pads were stored at room temperature for up to one month until analysis.

Urine samples over a 24 h period, and up to three days, were collected from willing participants of grain elevator A1 (wheat harvesting period) and grain elevator B. Each urination was collected separately, and the volume was recorded. Spot urine samples, including pre-shift and post-shift samples and first morning void on the following day, were collected from participants of grain elevator A2 (maize harvesting period), up to three days. The urine samples were stored after collection at −20 °C until analysis.

Urine collection was accompanied by a questionnaire for collecting contextual data on tasks performed in the previous day and during urine collection as well as the variety of grain handled, protective equipment and cereal-based food consumption.

Urinary creatinine was measured in urine samples using the Jaffé colorimetric method.

### 4.2. Determination of Mycotoxins in Urine and Air Samples

Quantification of mycotoxins in urine and air samples was performed using an earlier published approach [25]. The analytes of interest are presented in Table 6.

Briefly, urine samples were hydrolyzed overnight at 37 °C with glucuronidase enzyme to hydrolyze the glucuronide and/or sulfate conjugates of mycotoxins. For the determination of DON, hydrolyzed urine sample was loaded onto a solid phase extraction cartridge Oasis HLB (Waters, Milford, CT, USA) for further purification. The extracted sample was then analyzed by liquid chromatography–high-resolution mass spectrometry (LC-HR-MS/MS). For the determination of other mycotoxin biomarkers, an online Turboflow™ sample clean-up was performed before analysis by LC-HR-MS/MS. The samples were analyzed on a UHPLC–HR-MS/MS system. Mycotoxin separation was performed with an Accucore RP-MS column (150 × 2.1 mm, 2.6 µm, Thermo Scientific, San Francisco, CA, USA) at a temperature of 23 °C. The UHPLC system was coupled to a Q-Exactive™ benchtop mass spectrometer (Thermo Scientific) equipped with a heated electrospray ionization source (HESI II) operating in positive mode. The optimal MS parameters were S-Lens Radio Frequency (RF) level 50 operating with multiplexed events of targeted single ion monitoring (tSIM) and data-dependent fragmentations (ddMS2). Detection was performed for tSIM acquisition using a resolution of 70,000 FWHM. For ddMS2 acquisition, the resolution was 35,000 FWHM. The results are expressed in µg/L and in µg/g of creatinine. In this case, only urine samples with creatinine concentrations in the range of 0.5–3 g/L were considered.

For the determination of mycotoxins in air samples, the dust collected on the foam pad was weighted, then recovered with a mixture of a water/methanol. The whole sample extract was then diluted with a phosphate buffer saline solution, filtered through filter paper and applied to immunoaffinity columns AOF MS-PREP^®^ and DZT MS-PREP^®^ (R-BIOPHARM BiopharmRhône LTD, Saint-Didier-au-Mont-d’Or, France) connected in tandem. After elution, the sample was analyzed by high-performance liquid chromatography with fluorescence and UV detection, performed along with pre- or post-column derivatization as relevant to each mycotoxin. The results are expressed in mg.m^−3^ and ng.m^−3^ as the quantity of dust and mycotoxins collected on the foam pad and reported to the sampled air volume.

A description of the analytical procedure is detailed in the original publication of these methods [25].

### 4.3. Statistical Analysis

A descriptive analysis (reporting the minimum, median and maximum) was performed on urinary mycotoxin biomarkers and airborne mycotoxin concentrations for each elevator and by sampling time. Kinetic profile of urinary DON excretion was plotted for each worker as a function of the sampling time.

To compare DON, ZEN and OTA biomarker levels at different sampling times (with and without creatinine correction), a logarithmic transformation was applied to the DON, ZEN and OTA biomarker concentrations. A mixed linear regression model was used to test the “sampling times” fixed effect, including a “worker” random effect. When the “sampling time” effect was significant, a multiple comparisons post hoc test (with Bonferroni correction) was applied to test the differences of biomarkers levels between the different sampling times. The statistical significance threshold was set at 5%.

The correlation between post-shift urinary DON, ZEN and αZEL concentrations and airborne mycotoxin concentrations were estimated using the Pearson correlation coefficient on the log-transformed urinary and airborne mycotoxin concentrations. Statistical analyzes were performed using Stata Statistical Software (Version 16.1, StatCorp, College Station, TX, USA).

## Figures and Tables

**Table 1:** Characteristics of the study population.

	*n*	Age Median (Range)	Years of Activity Median (Range)	Smokers *n*
Grain elevator A1 (wheat harvest)	4	44 (31–55)	12 (1–19)	3
Grain elevator A2 (maize harvest)	8	44 (19–55)	5 (0.06–35)	6
Grain elevator B (maize harvest)	6	36.5 (26–56)	7.5 (0.7–18)	3

**Table 2:** Grain dust (mg.m^−3^) and mycotoxins levels (ng.m^−3^) in personal air samples from workers.

		Elevator A1 *n* ^1^ = 12	Elevator A2 *n* = 22	Elevator B *n* = 16	All Settings *n* = 50	Mycotoxin LOQ for 8 h Sampling (ng.m^−3^)
Grain dust	Median	4.2	4.2	7.9	4.9	
	Range	2.0–20.2	0–19.0	1.7–43.4	0–43.4	
DON	>LOQ ^2^ (*n*, (%))	2 (17%)	14 (64%)	11 (69%)	27 (54%)	
	Median ^3^	-	8.3	11.0	8.0	6.0
	Range	<LOQ–26.5	<LOQ–39.2	<LOQ–80.1	<LOQ–80.1	
ZEN	>LOQ (*n*, (%))	3 (25%)	20 (91%)	15 (94%)	38 (76%)	
	Median	-	11.0	79.5	12.0	1.0
	Range	<LOQ–13.9	<LOQ–90.0	<LOQ–778	<LOQ–778	
FB1	>LOQ (*n*, (%))	1 (8%)	19 (86%)	16 (100%)	36 (72%)	
	Median	-	13.8	28.6	13.1	6.0
	Range	<LOQ–13.5	<LOQ–170	6.8–248	<LOQ–248	
T-2	>LOQ (*n*, (%))	0	2 (9%)	2 (12%)	4 (8%)	
	Median	-	-	-	-	15.0
	Range	<LOQ	<LOQ–21.5	<LOQ–417	<LOQ–417	
HT-2	>LOQ (*n*, (%))	0	11 (50%)	13 (81%)	24 (48%)	
	Median	-	83.9	406	-	15.0
	Range	<LOQ	<LOQ–1263	<LOQ–2232	<LOQ–2232	

^1^*n*: number of samples collected; ^2^ LOQ: limit of quantification; ^3^ Median was not calculated when the proportion of measurements below the LOQ was >50%.

**Table 3:** Mycotoxin biomarker concentrations in urine samples from the three elevators.

		Elevator A1	Elevator A2	Elevator B	All Settings	LOQ
**µg/L**						
	***n* ^1^**	**45**	**69**	**81**	**195**	**µg/L**
DON	>LOQ ^2^ (*n* (%))	44 (98)	68 (99)	79 (98)	191 (98)	
	Median ^3^	14.60	16.80	13.30	14.50	0.05
	Range	<LOQ–66.40	<LOQ–154	<LOQ–72.80	<LOQ–154	
ZEN	>LOQ (*n* (%))	43 (98)	69 (100)	81 (100)	193 (99)	
	Median	0.11	0.17	0.12	0.13	0.025
	Range	<LOQ–0.29	0.03–0.56	0.03–1.0	<LOQ–1.0	
αZEL	>LOQ (*n* (%))	35 (78)	46 (67)	21 (26)	102 (52)	
	Median	2.16	1.73	<LOQ	1.14	1.00
	Range	<LOQ–48.9	<LOQ–7.62	<LOQ–2.17	<LOQ–48.9	
βZEL	>LOQ (*n* (%))	34 (76)	23 (23)	7 (9)	64 (33)	
	Median	0.90	<LOQ	<LOQ	<LOQ	0.50
	Range	<LOQ–3.72	<LOQ–1.67	<LOQ–1.02	<LOQ–3.72	
OTA	>LOQ (*n* (%))	36 (80)	65 (94)	47 (58)	148 (76)	
	Median	0.017	0.025	0.013	0.018	0.01
	Range	<LOQ–0.051	<LOQ–0.099	<LOQ–0.054	<LOQ–0.099	
T-2	>LOQ (*n* (%))	5 (12)	3 (5)	0 (0)	8 (4)	
	Median	<LOQ	<LOQ	<LOQ	<LOQ	1.00
	Range	<LOQ–2.65	<LOQ–0.96	<LOQ	<LOQ–2.65	
HT-2	>LOQ (*n* (%))	0 (0)	7 (10)	0 (0)	7 (4)	
	Median	<LOQ	<LOQ	<LOQ	<LOQ	0.50
	Range	<LOQ	<LOQ–5.63	<LOQ	<LOQ–5.63	
**µg/g creat**						
	***n* ^4^**	**45**	**65**	**60**	**170**	
DON	Median	9.27	11.90	15.20	12.50	
	Range	<LOQ–36.70	<LOQ–123	<LOQ–83.70	<LOQ–123	
ZEN	Median	0.07	0.12	0.12	0.11	
	Range	<LOQ–0.17	0.03–0.40	0.05–0.80	<LOQ–0.80	
αZEL	Median	1.11	1.26	-	0.87	
	Range	<LOQ–23.20	<LOQ–5.38	<LOQ–2.87	<LOQ–23.20	
βZEL	Median	0.44	-	-	-	
	Range	<LOQ–1.74	<LOQ–1.08	<LOQ–0.66	<LOQ–1.74	
OTA	Median	0.009	0.017	0.012	0.014	
	Range	<LOQ–0.026	<LOQ–0.052	<LOQ–0.041	<LOQ–0.052	
T-2	Median	-	-	-	-	
	Range	<LOQ–2.73	<LOQ–0.45	-	<LOQ–2.73	
HT-2	Median	-	-	-	-	
	Range	-	<LOQ–3.29	-	<LOQ–3.29	

^1^*n*: number of samples collected; ^2^ LOQ: limit of quantification; ^3^ Median was not calculated when the proportion of measurements below the LOQ was >50%; ^4^ *n*: number of measurements with creatinine value between 0.5 and 3 g/L.

**Table 4:** Urinary mycotoxin median concentrations (µg/L) in pre-shift, post-shift and first morning samples from the workers of the three elevators.

Mycotoxin/Sampling Time	Elevator A1		Elevator A2		Elevator B		All Settings	
	*n* ^1^ (%>LOQ)	Median ^2^	*n* (%>LOQ)	Median	*n* (%>LOQ)	Median	*n* (%>LOQ)	Median
DON (µg/L)								
Pre-shift samples	13 (92)	6.71	25 (100)	13.10	12 (100)	8.30	50 (98)	9.90
Post-shift samples	10 (100)	26.70	25 (100)	24.80	12 (100)	15.10	47 (100)	22.10
First morning samples	8 (100)	15.10	19 (95)	10.80	9 (100)	19.30	36 (97)	16.50
ZEN (µg/L)								
Pre-shift samples	13 (84)	0.11	25 (100)	0.14	12 (100)	0.11	50 (99)	0.12
Post-shift samples	10 (100)	0.08	25 (100)	0.21	12 (100)	0.15	47 (100)	0.16
First morning samples	8 (100)	0.09	19 (100)	0.14	9 (100)	0.14	36 (100)	0.14
αZEL (µg/L)								
Pre-shift samples	13 (77)	1.45	25 (68)	1.71	12 (8)	-	50 (56)	1.36
Post-shift samples	10 (80)	1.51	25 (72)	1.75	12 (42)	-	47 (66)	1.47
First morning samples	8 (75)	2.28	19 (58)	2.01	9 (22)	-	36 (52)	1.20
βZEL (µg/L)								
Pre-shift samples	13 (69)	0.98	25 (28)	-	12 (0)	-	50 (32)	-
Post-shift samples	10 (80)	1.11	25 (36)	-	12 (8)	-	47 (38)	-
First morning samples	8 (62)	0.79	19 (37)	-	9 (22)	-	36 (39)	-
OTA (µg/L)								
Pre-shift samples	13 (84)	0.017	25 (92)	0.022	12 (50)	0.010	50 (80)	0.020
Post-shift samples	10 (80)	0.015	25 (92)	0.024	12 (75)	0.024	47 (85)	0.023
First morning samples	8 (75)	0.024	19 (100)	0.027	9 (89)	0.016	36 (92)	0.024

^1^ number of samples; ^2^ Median was not calculated when the proportion of measurements below the LOQ was >50%.

**Table 5:** Urinary mycotoxin median concentrations (µg/g crea) in pre-shift, post-shift and first morning samples from the workers of the three elevators.

Mycotoxin/ Sampling Time	Elevator A1		Elevator A2		Elevator B		All Settings	
	*n*	Median	*n*	Median	*n*	Median	*n*	Median
DON (µg/g)								
Pre-shift samples	13	4.68	24	12.50	8	12.80	45	8.10
Post-shift samples	10	13.70	24	13.10	9	12.40	43	12.70
First morning samples	8	7.19	17	9.61	9	15.10	34	9.90
ZEN (µg/g)								
Pre-shift samples	13	0.08	24	0.12	8	0.14	45	0.12
Post-shift samples	10	0.06	24	0.13	9	0.13	43	0.13
First morning samples	8	0.06	17	0.09	9	0.11	34	0.09
αZEL (µg/g)								
Pre-shift samples	13	0.88	24	1.39	8	-	45	0.90
Post-shift samples	10	0.95	24	1.22	9	-	43	1.00
First morning samples	8	1.07	17	1.20	9	-	34	0.90
βZEL (µg/g)								
Pre-shift samples	13	0.51	24	-	8	-	45	-
Post-shift samples	10	0.52	24	-	9	-	43	-
First morning samples	8	0.40	17	-	9	-	34	-
OTA (µg/g)								
Pre-shift samples	13	0.005	24	0.012	8	0.013	45	0.016
Post-shift samples	10	0.014	24	0.013	9	0.012	43	0.016
First morning samples	8	0.007	17	0.010	9	0.015	34	0.013

**Table 6:** Mycotoxins of interest and LOQ values in urine and air samples.

Mycotoxin Biomarkers in Urine Samples	LOQ * µg/L	Mycotoxin in Air Samples	LOQ for 8 h Sampling (ng.m^−3^)
AFB1	0.01	AFB1	0.06
AFM1	0.05	DON	6
DON	0.05	FB1	6
FB1	0.25	OTA	0.014
OTA	0.01	ZEN	1
OTα	0.25	T-2	15
ZEN	0.025	HT-2	15
αZEL	1		
βZEL	0.5		
T-2	0.5		
HT-2	1		

* LOQ: limit of quantification.

## Data Availability

The data presented in this study are available on request from the corresponding author. The data are not publicly available due to company privacy.
